# A Laboratory Assessment of Factors That Affect Bacterial Adhesion to Contact Lenses

**DOI:** 10.3390/biology2041268

**Published:** 2013-11-01

**Authors:** Debarun Dutta, Mark DP Willcox

**Affiliations:** 1 School of Optometry and Vision Science, The University of New South Wales, Sydney NSW 2052, Australia; 2 Brien Holden Vision Institute, Sydney NSW 2052, Australia; E-Mail: m.willcox@unsw.edu.au

**Keywords:** Bacterial adhesion, contact lens, *Pseudomonas aeruginosa*, *Staphylococcus aureus*

## Abstract

Adhesion of pathogenic microbes, particularly bacteria, to contact lenses is implicated in contact lens related microbial adverse events. Various *in vitro* conditions such as type of bacteria, the size of initial inoculum, contact lens material, nutritional content of media, and incubation period can influence bacterial adhesion to contact lenses and the current study investigated the effect of these conditions on bacterial adhesion to contact lenses. There was no significant difference in numbers of bacteria that adhered to hydrogel etafilcon A or silicone hydrogel senofilcon A contact lenses. *Pseudomonas aeruginosa* adhered in higher numbers compared to *Staphylococcus aureus*. Within a genera/species, adhesion of different bacterial strains did not differ appreciably. The size of initial inoculum, nutritional content of media, and incubation period played significant roles in bacterial adhesion to lenses. A set of *in vitro* assay conditions to help standardize adhesion between studies have been recommended.

## 1. Introduction

Contact lenses provide several benefits over spectacles, but their wear has remained as a risk factor for the development of various adverse events, such as microbial keratitis (MK) [1], contact lens related acute red eye (CLARE) [2], contact lens peripheral ulcer (CLPU) [3] and infiltrative keratitis (IK) [4]. Adhesion and colonization by variety of microbes, particularly bacteria [1], to contact lenses is implicated as a major factor in the initiation of these adverse events. *Pseudomonas aeruginosa* and *Staphylococcus aureus* are the two predominant microorganisms implicated in contact lens related microbial adverse events [1,5] and other microorganisms such as *Serratia marcescens* [2], coagulase-negative *staphylococci* [1], fungus [6] and *Acanthamoeba* [7] are less frequently involved. Depending on the study design and location, *P. aeruginosa* and *S. aureus* together account 44% to 57% of total culture positive contact lens related microbial keratitis [1,8]. 

Bacterial adhesion to contact lenses is a complex and multifactorial process and previous *in vitro* and *ex vivo* adhesion data differ widely between various studies [9]. This is mainly due to variety of methodology used to evaluate bacterial adhesion and there are a range of assay conditions that have been used to evaluate bacterial adhesion to lenses. These conditions have included different strains/types of bacteria, contact lenses types, inoculum sizes, the nutritional content of media and the incubation time for adhesion to occur [9]. Viable plate count [10,11,12], number of cells adherent to parallel plate flow chambers [13], scanning electron microscopy [14], bioluminescent ATP assay [15], light microscopy [16], and assessment of the number of cells after radio-labeling [17] have been used to quantify microbial adhesion to lenses. Various solutions are used during adhesion experiments which include phosphate buffer saline (PBS) [18,19], which is nutritionally inert, and broths such as Tryptone Soy [20] or Mueller Hinton which are nutritionally rich. The reported inoculum sizes in bacterial adhesion assays have varied from 1 × 10^3^ colony forming units (CFU) mL^−1^ up to 1 × 10^9^ CFU mL^−1^ [10,21] and the incubation period for adhesion has ranged from 10 minutes to 72 hours [16,22].

The wide variety of bacterial assays used in previous studies and consequent differences in bacterial numbers adhering to lenses, signify a need to develop a set of standardized *in vitro* assay that can allow comparisons within and between studies on adhesion of different bacterial strains to different contact lenses. This study aimed for a better understanding of these major influencing factors that affect bacterial attachment and furthermore suggest key standard assay conditions that are best suited for laboratory assessment. As biofilm formation on contact lenses during wear is infrequent [23] the primary focus of this investigation was on initial steps in bacterial adhesion. 

## 2. Experimental Section

Two of the most widely used contact lens materials, the hydrogel etafilcon A (ACUVUE® 2; Johnson & Johnson Vision Care Inc., Jacksonville, FL; Base curve: 8.7 mm, Diameter: 14.0 mm, Power: −3.00 Diopter) and the silicone hydrogel senofilcon A (ACUVUE® OASYS™; Johnson & Johnson Vision Care; Base curve: 8.4 mm, Diameter: 14 mm, Power: −3.00 Diopter) were used [24]. The properties of these materials are described in Table 1.

**Table 1:** Properties of contact lens materials evaluated in the study.

Proprietary name	ACUVUE® 2	ACUVUE® OASYS™
United States Adopted Name (USAN)	etafilcon A	senofilcon A
Manufacturer	Johnson & Johnson	Johnson & Johnson
Water content (%)	58	38
Oxygen Permeability (Dk)	21	103
Centre thickness (mm) - 3.00 Ds	0.08	0.07
Oxygen Transmissibility (Dk/t) at 35 °C	25	147
FDA group	IV	I
Surface treatment	None	No surface treatment. Internal wetting agent (PVP) that also coats the surface
Principal monomers	HEMA + MA	mPDMS + DMA + HEMA + siloxane macromer + PVP + TEGDMA

mPDMS, (monofunctional polydimethylsiloxane); DMA, (N,N-dimethylacrylamide); HEMA, (2-hydroxyethyl methacrylate); PVP, (polyvinyl pyrrolidone); TEGDMA (tetraethyleneglycol dimethacrylate); MA, (methacrylic acid).

### 2.1. Bacterial Strains

As the majority of the causative microorganisms for contact lens related microbial adverse events are Gram negative *Pseudomonas aeruginosa* and Gram positive *Staphylococcus aureus* [1,5], selected strains of these were used. Table 2 details the bacterial strains used in this study [19,25,26,27,28].

**Table 2:** Details of bacteria used in the study.

***S. aureus* strains**	**Isolation site**
*S. aureus* 31 [26]	CLPU – contact lens
*S. aureus* 38 [26]	MK
***P. aeruginosa* strains**	**Isolation site**
*P. aeruginosa* 6294 [25]	MK
*P. aeruginosa* ATCC 9027 [27]	Otic infection
*P. aeruginosa* GSU3 [19,28]	Human Corneal Ulcer

### 2.2. Assay Media

Four different types of bacterial suspension media, phosphate buffered saline pH 7.4 (PBS; NaCl 8 g L^−1^, KCl 0.2 g L^−1^, Na_2_HPO_4_ 1.15 g L^−1^, KH_2_PO_4_ 0.2 g L^−1^), tryptone soy broth (TSB; Oxoid, Basingstoke, UK), TSB diluted 10X in sterile PBS (1/10 TSB), or 1/10 TSB containing glucose (0.25% w/v) (TSBG) were used. PBS acted as a nutritionally inert media and TSB as a highly nutritious media.

### 2.3. Incubation Period

Contact lenses were incubated for two hours and 18 hours with the bacterial suspensions.

### 2.4. Inoculum Size

1 × 10^3^ CFU mL^−1^, 1 × 10^6^ CFU mL^−1^ and 1 × 10^10^ CFU mL^−1^ are the three inoculum sizes used in this study. 

### 2.5. Adhesion Conditions

Stock cultures were stored in 30% glycerol at −80 °C. Bacteria were grown overnight in TSB at 37 °C with aeration. The harvested bacterial cells were centrifuged for 10 mins at 3,000 rpm and the cells washed three times with PBS. All the bacteria were then resuspended in one of the four media to an OD_660nm_ of 1.0 (1 × 10^9^ CFU mL^−1^). The bacterial cell suspensions were then diluted to 1 × 10^6^ and 1 × 10^3^. The bacterial suspension of 1 × 10^10^ CFU mL^−1^ was made by centrifuging 10 mL of 1 × 10^9^ CFU mL^−1^ and resuspending it in 1 mL respective media. Contact lenses were washed three times in PBS and transferred to 1 mL of bacterial suspensions in wells of 24-well tissue culture plates (CELESTAR®, Greiner bio-one, Frickenhausen, Germany), concave side up. To allow adhesion of bacterial cells, lenses were incubated for two hours or 18 hours at 37 °C with shaking (120 rpm). Lenses were aseptically removed from the suspension and washed three times with 1 mL PBS in a 24-well plate by shaking at 120 rpm for 30 seconds to remove non-adherent cells. Following washing, contact lenses were stirred rapidly in 2 mL of PBS containing a small magnetic stirring bar. Following log_10_ serial dilutions in PBS, 3 × 50 µL of each dilution were plated on a nutrient agar (NA; Oxoid, Basingstoke, UK). After 24 hours incubation at 37 °C, the viable bacteria were enumerated as CFU/lens mm^2^. The inoculum sizes were retrospectively counted by plating and overnight incubation on nutrient agar. Results are expressed as the numbers of adherent viable bacteria from three independent experiments with three samples evaluated each time.

### 2.6. Statistics

The adhesion data were log_10_ (x+1) transformed prior to data analysis where x is the number of adherent bacterial colonies mm^−2^. All data were analyzed using Statistical Package for Social Science for Windows version 21.0 (SPSS, Inc, Chicago, IL). Interactions between different factors influencing bacterial adhesion to contact lenses such as bacterial strain type, assay media, incubation time and inoculum size were investigated in a nested model of all the variables. Based on this estimation, by factoring all the variables, the estimated mean was calculated which is adjusted for the other variables in the model. To evaluate and compare the influence of tested assay conditions on bacterial adhesion, partial Eta squared was estimated. Bacterial adhesion and contact lens parameters were analyzed using independent two sample *t* test. Differences between the groups were analyzed using a linear mixed model ANOVA, which adjusts for the correlation due to repeated observations. Post hoc multiple comparisons were done using Bonferroni correction. Statistical significance was set at 5%. 

## 3. Results and Discussion

**Figure 1:**
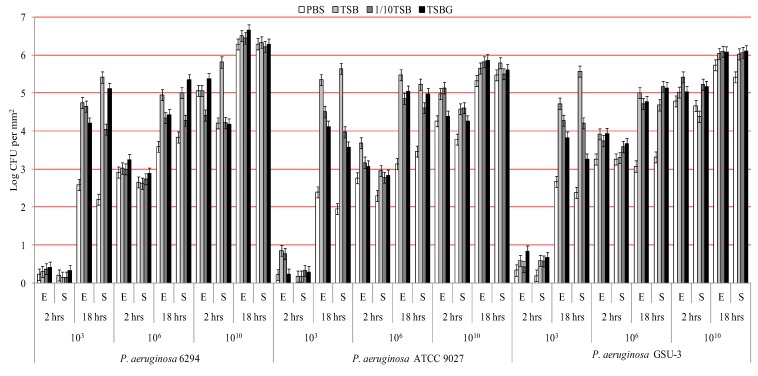
Adhesion of *P. aeruginosa* to contact lenses under different conditions (E = etafilcon A lenses; S = senofilcon A lenses).

**Figure 2:**
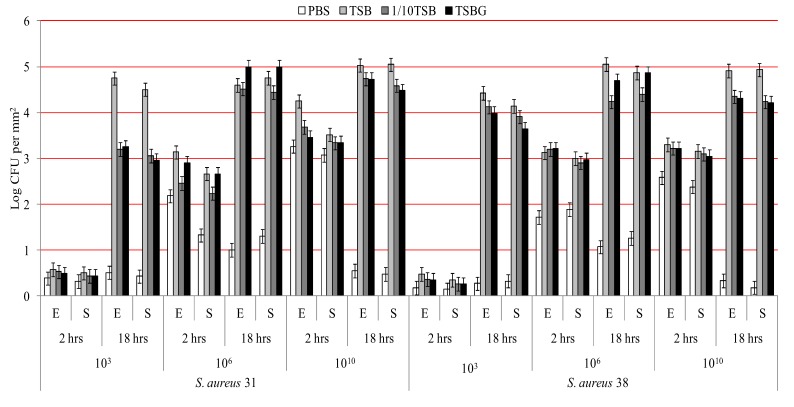
Adhesion of S*. aureus* to contact lenses under different conditions. (E = etafilcon A lenses; S = senofilcon A lenses).

Figure 1 and Figure 2 show the adhesion of *P. aeruginosa* and *S. aureus* respectively when incubated in the four different media and at three different bacterial concentrations over time. Analysis of strain differences within a genera/species found that only *P. aeruginosa* ATCC 9027 showed higher adhesion to etafilcon A than senofilcon A (*p* < 0.01) and not for any other bacterial type. *P. aeruginosa* adhered at higher number compared to *S. aureus* (*p* < 0.01).

For each bacterial type and strain there was a significant increase in adhesion from 2 to 18 hours (*p* < 0.01) when incubated with 1 × 10^3^ CFU mL^−1^ or 1 × 10^6^ CFU mL^−1^ bacterial suspension. For *P. aeruginosa* strains, adhesion to the contact lenses increased as the initial inoculum increased (*p* < 0.01). However, for strains of *S. aureus* adhesion reached a maximum when 1 × 10^6^ CFU mL^−1^ bacterial cells were incubated with lenses; addition of bacteria at 1 × 10^10^ CFU mL^−1^ did not increase adhesion. The differences between the number of bacterial cells recovered from the washed solutions of the contact lenses incubated with different concentrations of bacteria was less than 0.3 log.

When comparing the effect of different media on adhesion, there were differences between the bacterial genera/species. For *P. aeruginosa*, adhesion was significantly lower (*p* < 0.01) when incubated in PBS after 18 hours for concentrations up to and including 1 × 10^6^ CFU mL^−1^, but not at 1 × 10^10^ CFU mL^−1^. At 1 × 10^3^ CFU mL^−1^ adhesion of *P. aeruginosa* was significantly higher when incubated with TSB (*p* < 0.01) compared to all other media, but this difference tended to lose significance at higher bacterial concentrations. For *S. aureus*, adhesion was significantly lower in PBS (*p* < 0.01) than all other media at all bacterial concentrations, at all time points and on both contact lens types. When 1 × 10^6^ or 1 × 10^10^ CFU mL^−1^ of *S. aureus* was used, there was a reduction in bacterial numbers adhered to lenses when incubated in PBS after 18h adhesion compared to 2 hours adhesion; this was not the case with other media. 

After adjusting for effects of incubation time, inoculum size and lens material, incubation with PBS showed significantly (*p* < 0.01) less adhesion for all the bacteria studied. There were no significant differences in bacterial adhesion (*p* > 0.05) when incubated with 1/10 TSB or TSBG. Incubation in the nutritionally rich TSB was often associated with higher adhesion (Figure 1 and Figure 2) compared to other media especially after 18 hours.

Table 3 shows the estimated degree of association between bacterial adhesion and influencing assay conditions. Higher partial Eta squared value implies higher influence over bacterial adhesion. Variation in *S. aureus* strains did not influence bacterial adhesion (partial Eta squatted = 0.00; *p* = 0.41). Rest all the factors including various *P. aeruginosa* strains, lens types, assay media, incubation period and inoculum size had significant influence (*p* < 0.05) on *P. aeruginosa* and *S. aureus* adhesion.

**Table 3:** Effect size of factors that influence bacterial adhesion.

Influencing factor for bacterial adhesion	Partial Eta squared	
	*P. aeruginosa*	*S. aureus*
Inoculum size	0.75	0.43
Incubation period	0.64	0.37
Assay media	0.19	0.54
Type of lens	0.01	0.01
Bacterial strains	0.02	0.00

In this study, adhesion of *P. aeruginosa* or *S. aureus* strains to contact lenses was assessed under several assay conditions. In most cases there was no significant difference in adhesion to hydrogel etafilcon A or silicone hydrogel senofilcon A lenses, which is consistent to some earlier studies [19]. However, this result was somewhat different to studies showing higher *P. aeruginosa* and *S. aureus* adhesion to silicone hydrogel contact lenses compared to hydrogel lenses [10,15,29]. Senofilcon A lenses have been shown to result in lower bacterial adhesion compared to other silicone hydrogels such as balafilcon A or lotrafilcon B used in previous studies [30]. 

Different strains of *P. aeruginosa* or *S. aureus* did not have significantly different adhesion to contact lenses. Previous studies have shown considerable variation in adhesion between different strains of *P. aeruginosa* or *S. aureus,* ranging up to 2.00 × 10^5^ CFU mm^−2^ and 1.23 × 10^5^ CFU mm^−2^ respectively [19,31,32,33]. Thus it is important to use the same strains across studies for meaningful comparisons to be made. Other strains can be incorporated as well to test for strain differences.

*P. aeruginosa* adhered at higher levels than *S. aureus* and this is in agreement with the previous reports [19,34]. However, the reason is not known in any great detail. It is known that cell surface appendages such as flagella and pili aid in the adhesion of *P. aeruginosa* [35] as does the relatively hydrophobic nature of some strains of *P. aeruginosa* compared to *S. aureus* [36]. This finding has been hypothesized to be a reason for the finding that *P. aeruginosa* is a predominant causative agent in contact lens induced-MK.

Previous studies have elucidated that the initial bacterial adhesion to contact lenses increases with time, peaked at 3 to 18 hours of incubation and then remained steady, suggesting the end point of primary adhesion [22,31,37]. Bacterial adhesion during two phases of the process, two hours and 18 hours exposure of contact lenses to bacterial suspension were determined in this study. The viable bacterial numbers after 18 hours adhesion were generally higher compared with after 2 hours, an observation that agrees with some previous studies [22,38]. Combining our results with Tran *et al*. [35] showing linear kinetics of bacterial adhesion up to 70 minutes and Randler *et al*. [22] investigating up to 72 hours but having incremental adhesion only up to 24 hours, illustrates that adhesion to contact lenses increases in a time dependant manner up to 18-24 hours of incubation and then viability is reduced. Perhaps, the reduction in viability is due to the bacteria entering a biofilm mode of growth, which is known to result in lower viability of cells [39,40] or due to biofilm dispersal that can occur when the environment nutrients are not favorable for bacteria. In contrast, Stapleton *et al.* [41] and Andrews *et al.* [37] reported a plateau in adhesion that was reached after 45 minutes and four hours incubation respectively, with the adhesion that remained at those levels for more than 18 hours. These findings illustrates that investigators need to select incubation period of a bacterial adhesion carefully, depending upon study hypothesis being tested.

Bacterial incubation in the nutritionally rich media TSB resulted in the highest adhesion of both bacterial types. PBS, being nutritionally inert, resulted in apparent death or the more fastidious *S. aureus* strains used in the current study, and so PBS is not recommended as a media for adhesion experiments. This fact was supported by a test showing 18 hours incubation of 1 × 10^3^ CFU mL^−1^, 1 × 10^6^ CFU mL^−1^ and 1 × 10^10^ CFU mL^−1^ significantly (*p* < 0.001) reduced mean *S. aureus* viability to 2.13 × 10^2 ^CFU mL^−1^, 2.55 × 10^3^ CFU mL^−1^ and 9.13 × 10^4^ CFU mL^−1^ in PBS (data not shown). This study demonstrates that diluted TSB can function as an adequate media for adhesion experiments. Since there was no significant difference in bacterial adhesion with 1/10 TSB and TSBG, addition of glucose is not recommended. 

Since it is difficult to quantify exposure of contact lenses to microorganisms during wear, a wide range of numbers were selected for testing; 1 × 10^3^ CFU mL^−1^ represented a low inoculum size, 1 × 10^6^ CFU mL^−1^ a medium inoculum size and 1 × 10^10^ CFU mL^−1^ represented very high inoculum size. 1 × 10^10^ CFU mL^−1^ was usually associated with highest adhesion, especially when incubated for 2 hours. Previous studies have also used higher inoculum sizes when incubation times were short [41,42,43] and a lower inoculum size when incubated for longer [20,21]. Contact lenses will rarely be exposed to such high numbers of bacteria such as 1 × 10^10 ^CFU mL^−1^ during contact lens wear or even in lens cases. The range of bacterial numbers isolated from contact storage lens storage cases has been reported to be 1.24 × 10^4^ CFU/case to 6.32 × 10^4^ CFU/case [44,45,46,47,48,49]. Therefore, exposing contact lenses to this level of bacteria may be unrealistic. The data from the current experiments suggest that an inoculum size of 1 × 10^6^ CFU mL^−1^ may offer a more realistic level of bacteria to expose contact lenses to, and results in medium to high levels of bacterial adhesion. 

Inoculum size was the greatest influencing factor for *P. aeruginosa* adhesion, followed by incubation period and assay media. Interestingly, nutritionally variable assay media was the greatest influencing factor determining *S. aureus* adhesion, confirming that *S. aureus* is sensitive to the nutritional content. Incubation period and inoculum size were the other major influencing factors. Lens types and bacterial strains had a minor influence. 

A limitation of this study is that bacterial adhesion to contact lens was not evaluated at frequent time intervals, which might have provided better understanding regarding kinetics of bacterial adhesion. Bacterial adhesion after longer incubation period such as 18 hours is complex procedure because of the bacteria are more likely to be replicating during this time, especially under nutrient enhanced conditions, probably combinations of initial biofilm formation and continued initial adhesion of daughter cells. This study has evaluated adhesion at a fixed stirring rate (120 rpm), thus altering the rate will undoubtedly implicate the rate of bacterial arrival to lenses. Since, it is difficult to reproduce *in vivo* blinking motion onto contact lens surfaces *in vitro*, we would recommend using a constant shaking rate (such as used in this study; 120 rpm) for a particular study design. In addition, total microbial load cannot be investigated by this type of assay. However, viable plate count is a vital method to evaluate reproducible microbial count, essential for development of infection and inflammation especially at the ocular environment [3]. Based on the results obtained in this study we suggest 18 hours incubation of 10^6^ CFU mL^−1^ *S. aureus* or *P. aeruginosa* in 1/10 TSB or PBS respectively to study the attachment of bacteria to contact lenses. The advantages of this recommended assay also include that better results could be achieved with the use of basic laboratory apparatuses and does not require expensive machines such confocal or optical microscope and microtitre plate reader. The bacterial adhesion assay used in this study suits best to investigate increase or decrease in viable count such as used in antimicrobial research. 

It is important to carefully select assay conditions depending on the study purpose. Adhesion of *P. aeruginosa* to contact lenses ranged from 1.38 CFU mm^−2^ to 4.57 × 10^6^ CFU mm^−2 ^and *S. aureus* adhesion ranged from 1.37 CFU mm^−2 ^to 1.13 × 10^5^ CFU mm^−2^, depending on the assay conditions. If experiments are designed to investigate effect of materials on bacterial adhesion, or whether antimicrobial lenses can reduce adhesion, it is important that assay conditions are chosen that allow adhesion to control lenses at a medium range so that increases or decreases in adhesion can be measured. A set of such assay conditions is given in Table 4 that have provided moderate adhesion of between 1 × 10^3^ CFU mm^−2^ to 1 × 10^5 ^CFU mm^−2^ for *P. aeruginosa* and 1 × 10^3^ CFU mm^−2^ to 1 × 10^4.5^ CFU mm^−2^ for *S. aureus* for both contact lens types. 

**Table 4:** List of assay conditions with estimated mean adhesion 1 × 10^3^ CFU mm^−2^ to 1 × 10^5^ CFU mm^−2^ for *P. aeruginosa* and 1 × 10^3^ CFU mm^−2^ to 1 × 10^4.5^ CFU mm^−2^ for *S. aureus* for both etafilcon A and senofilcon A contact lens.

Bacteria*	Inoculum size (CFU mL^−1^)	Media	Incubation time (hours)	etafilcon A			senofilcon A		
				Estimated mean (CFU mm^-2^)	95% Confidence Interval (CFU mm^−2^)		Estimated mean (CFU mm^−2^)	95% Confidence Interval (CFU mm^−2^)	
					Lower Bound	Upper Bound		Lower Bound	Upper Bound
*P aeruginosa* 6294	10^10^	1/10 TSB	2	4.4	4.3	4.6	4.2	4.1	4.4
	10^6^	1/10 TSB	18	4.3	4.2	4.5	4.3	4.1	4.4
	10^6^	PBS	18	3.6	3.4	3.7	3.8	3.7	4.0
	10^3^	1/10 TSB	18	4.7	4.5	4.8	4.0	3.9	4.2
*P. aeruginosa* ATCC 9027	10^10^	TSBG	2	4.4	4.2	4.5	4.3	4.1	4.4
	10^10^	PBS	2	4.3	4.1	4.4	3.8	3.6	3.9
	10^6^	1/10 TSB	18	4.9	4.7	5.0	4.6	4.5	4.8
	10^6^	PBS	18	3.1	3.0	3.3	3.5	3.3	3.6
	10^3^	1/10 TSB	18	4.5	4.4	4.7	4.0	3.8	4.1
	10^3^	TSBG	18	4.1	4.0	4.3	3.6	3.4	3.7
*P. aeruginosa* GSU-3	10^10^	PBS	2	4.8	4.6	4.9	4.7	4.5	4.8
	10^6^	1/10 TSB	2	3.7	3.6	3.9	3.6	3.4	3.7
	10^6^	TSBG	2	3.9	3.8	4.1	3.7	3.5	3.8
	10^6^	PBS	2	3.3	3.1	3.4	3.3	3.1	3.4
	10^6^	PBS	18	3.1	2.9	3.2	3.3	3.2	3.5
	10^6^	TSB	2	3.9	3.8	4.1	3.3	3.2	3.4
	10^6^	1/10 TSB	18	4.3	4.1	4.4	4.2	4.1	4.4
	10^3^	TSBG	18	3.8	3.7	4.0	3.3	3.1	3.4
*S. aureus* 31	10^10^	1/10 TSB	2	3.7	3.5	3.8	3.3	3.2	3.5
	10^10^	TSBG	2	3.5	3.3	3.6	3.3	3.2	3.5
	10^10^	TSB	2	4.2	4.1	4.4	3.5	3.4	3.7
	10^10^	TSB	2	4.2	4.1	4.4	3.5	3.4	3.7
	10^6^	1/10 TSB	18	4.5	4.4	4.7	4.4	4.3	4.6
*S. aureus* 38	10^10^	1/10 TSB	18	4.3	4.2	4.5	4.2	4.1	4.4
	10^10^	TSBG	18	4.3	4.2	4.5	4.2	4.1	4.4
	10^10^	TSB	2	3.3	3.2	3.4	3.2	3.0	3.3
	10^6^	1/10 TSB	18	4.2	4.1	4.4	4.4	4.3	4.5
	10^3^	1/10 TSB	18	4.1	4.0	4.3	3.9	3.8	4.0
	10^3^	TSB	18	4.4	4.3	4.6	4.1	4.0	4.3
	10^3^	TSBG	18	4.0	3.8	4.1	3.6	3.5	3.8

In conclusion, this study has determined that different strains of *P. aeruginosa* or *S. aureus* do not adhere very differently to contact lenses. Adhesion is more affected by the environment and numbers of bacteria initially applied to lenses. At least for etafilcon A and senofilcon A lenses, adhesion was not affected by lens polymer type. There are varieties of ingredients used to evaluate bacterial adhesion and investigators are required to select a set of bacterial assay depending on the study hypothesis. The proposed conditions that give intermediate levels of bacterial adhesion to contact lenses could be used for subsequent evaluation of bacterial adhesion to lenses or antibacterial efficacy of contact lenses.
